# Preclinical Assessment of IgY Antibodies Against Recombinant SARS-CoV-2 RBD Protein for Prophylaxis and Post-Infection Treatment of COVID-19

**DOI:** 10.3389/fimmu.2022.881604

**Published:** 2022-05-10

**Authors:** Andres Agurto-Arteaga, Astrid Poma-Acevedo, Dora Rios-Matos, Ricardo Choque-Guevara, Ricardo Montesinos-Millán, Ángela Montalván, Gisela Isasi-Rivas, Yudith Cauna-Orocollo, María de Grecia Cauti-Mendoza, Norma Pérez-Martínez, Kristel Gutierrez-Manchay, Ingrid Ramirez-Ortiz, Dennis Núñez-Fernández, Mario I. Salguedo-Bohorquez, Stefany Quiñones-Garcia, Manolo Fernández Díaz, Luis A. Guevara Sarmiento, Mirko Zimic, COVID-19 Working Group in Perú, Andres Agurto-Arteaga

**Affiliations:** ^1^ Laboratorio de Biotecnología Molecular y Genómica, Laboratorios de Investigación y Desarrollo, Farmacológicos Veterinarios SAC (FARVET SAC), Chincha, Peru; ^2^ Laboratorio de Bioinformática, Biología Molecular y Desarrollos Tecnológicos, Laboratorios de Investigación y Desarrollo, Facultad de Ciencias y Filosofía, Universidad Peruana Cayetano Heredia, Lima, Peru

**Keywords:** SARS-CoV-2, COVID-19, egg-yolk antibodies, IgY, passive immunization, receptor binding domain

## Abstract

Within the framework of the current COVID-19 pandemic, there is a race against time to find therapies for the outbreak to be controlled. Since vaccines are still tedious to develop and partially available for low-income countries, passive immunity based on egg-yolk antibodies (IgY) is presented as a suitable approach to preclude potential death of infected patients, based on its high specificity/avidity/production yield, cost-effective manufacture, and ease of administration. In the present study, IgY antibodies against a recombinant RBD protein of SARS-CoV-2 were produced in specific-pathogen-free chickens and purified from eggs using a biocompatible method. *In vitro* immunoreactivity was tested, finding high recognition and neutralization values. Safety was also demonstrated prior to efficacy evaluation, in which body weight, kinematics, and histopathological assessments of hamsters challenged with SARS-CoV-2 were performed, showing a protective effect administering IgY intranasally both as a prophylactic treatment or a post-infection treatment. The results of this study showed that intranasally delivered IgY has the potential to both aid in prevention and in overcoming COVID-19 infection, which should be very useful to control the advance of the current pandemic and the associated mortality.

## Introduction

COVID-19 is a potentially fatal infectious disease that rapidly spread through contact between infected people and surfaces which has made it a pandemic by the World Health Organization. To date, more than 483 million confirmed cases and 6.13 million deaths have been reported (1), significantly affecting the economic activities and lifestyles worldwide (2).

The virus responsible for this disease, SARS-CoV-2, is a betacoronavirus closely related to SARS-CoV (3), both with the same viral entry mechanism (4) relying on Spike (S) protein and its Receptor Binding Domain (RBD), which is essential to mediate the binding of S protein to human ACE2 receptor (5, 6). Hence, S and RBD are the main therapeutic targets, for which there are several vaccine and drugs candidates currently being clinically evaluated as countermeasures against COVID-19.

Vaccines based on inactivated viruses, mRNA, and those using viral vectors (7–9) have been applied to control the outbreak, however, there are major issues to overcome, such as the time-consuming process of development (10), SARS-CoV-2 variants escaping vaccine-induced antibodies appearing over time (11, 12), vaccine hesitancy reaching more than 40% in several countries and affecting adults and children through parental attitude (13, 14), and immunocompromised people in which seroconversion rates are significantly lower (15). Moreover, there is restricted access to vaccines in developing countries due to the control exercised by patent holders and pharmaceutical companies over prices (16).

In that regard, treatment by passive immunization could be a suitable option as an aid to vaccination, since it has had an important role in the control of multiple diseases, including the pandemic caused by the H1N1 influenza virus (17–19). This approach has already been applied in severe COVID-19 patients where plasma from convalescent patients was used, leading to the reduction of viremia and a substantial clinical improvement (20, 21).

Thereby, anti-SARS-CoV-2 IgG antibodies have significant therapeutic potential, however, there are some difficulties such as the low amount of specific antibodies recovered from a patient, and the possible adverse reactions that could present as an increase in pro-inflammatory disease mediated by antibodies known as antibody-dependent enhancement (ADE), or exacerbated endocytosis or viral phagocytosis in host cells through Fc receptors due to the presence of non-neutralizing antibodies, which enhances viral replication (22).

On the other hand, the therapeutic potential of IgY (yolk immunoglobulins) has been widely reviewed over the years, being used both in treatment and prevention of multiple respiratory diseases (23, 24), and proving to be a promising therapeutic method of passive immunity since IgY does not activate human complement nor induce allergic response in most of the population, granting safeness when administered in mammals, also reporting great stability in a wide range of temperature and pH conditions (25, 26).

Here, anti-RBD IgY antibodies were produced by immunizing SPF hens with a recombinant RBD protein, and *in vitro* immunoreactivity was tested. Subsequently, a challenge assay using SARS-CoV-2 was performed to evaluate the efficacy of IgY both as prophylactic and post-infection treatments.

## Materials and Methods

### Animals

Thirty-five-week-old White Leghorn hens from the Farvet SPF Hatchery were used for IgY production. Seven-week-old BALB/c female mice were purchased from the Universidad Peruana Cayetano Heredia and used for safety studies. Seven-week-old male and female golden Syrian hamsters were purchased from the National Institutes of Health and used throughout the efficacy assessment. All animals were acclimatized for a minimum of 1 week at the Veterinary Center’s biosafety level 2 containment facility of Farvet before experimental manipulation and provided sterilized water and food *ad libitum.*

### Ethics Statement

All procedures involving animal handling were approved by the Bioethics Committee of the Universidad Nacional Hermilio Valdizán registered as approval certificates of Research Project No. 1, 2, and 10. Animal immunizations and procedures were performed by qualified personnel following the ARRIVE guidelines (27). All the *in vivo* hamster experiments involving infectious SARS-CoV-2 were conducted under an appropriate biosafety level 3 laboratory (BSL3), at Farvet, Perú.

### IgY-R Elicitation in Hens

For the recombinant RBD protein of SARS-CoV-2 (rRBD) production, the Pro330-Ser530 region was extracted from the SARS-CoV-2 reference genome Wuhan-Hu-1 (Genbank accession number: NC_045512.2), for the RBD construct design, containing a gp67 secretion signal peptide at the N-terminus and a 10x-His tail at the C-terminus, and further produced in Sf9 insect cells through the baculovirus expression system approach, as described elsewhere (28). Four different amounts of the purified RBD protein (5, 12.5, 25, or 50 µg) and an aliquot of PBS for control were emulsified 1:1 with Montanide™ Seppic ISA 71 R VG-36518T adjuvant and administered intramuscularly at the pectoral muscle to SPF hens (n = 2 per group) every 2 weeks, for a total of three doses. Eggs were collected from immunized hens each week for immunoreactivity evaluation throughout 10 weeks post-vaccination (WPV).

### IgY-R Purification

The purification of the IgY-R was carried out following the biocompatible method proposed by Hodek (29) and modified by Wibawan (30), consisting of preparation of a water-soluble fraction (WSF) of yolk and saline precipitation of IgY. Briefly, the egg yolk was solubilized by diluting it seven times with distilled water and adjusting the pH to 5.0 with 0.5 M HCl. The mixture was frozen at -20°C and later thawed at 4°C. The yolk granules were pelleted by centrifugation at 13.500 g for 15 min at 4°C, the supernatant (WSF) was filtered and solid NaCl was added up to a concentration of 8.8%. Subsequently, it was stirred for 2 hours at room temperature following a pH adjustment to 4 with 0.5 M HCl, and then centrifuged at 3700 g for 20 min at 4°C. The supernatants were discarded, and the pellet resuspended in phosphate buffer (pH 7.0). The resulting IgY solution was dialyzed against phosphate buffer overnight and concentration was determined using the Bradford Reagent (Sigma) prior to being stored at 4°C until use.

### SDS-PAGE

To assess purity, a polyacrylamide gel electrophoresis in presence of sodium dodecyl sulfate (SDS-PAGE) under reducing conditions was performed, in a Mini-PROTEAN Tetra Cell (Biorad), mixing samples with sample buffer and loading 20 µL of samples and 5 µL of the Broad Multi Color Pre-Stained Protein Standard (Genscript) into the wells of ExpressPlus PAGE Gels (Genscript) and using Tris-MOPS-SDS buffer (Genscript) as running buffer, in a two-step running starting at 60V for 30 min and then changed to 110 V for 1 hour and 30 minutes, with a PowerPac Basic power supply (Biorad). Gels were stained with 0.25% Coomassie Blue R-250 for 4 hours and distained with a solution containing 10% acetic acid, 30% methanol, and 60% water, applying four washes of 30 min each. Both staining and distaining were performed using a rocking platform settled at 14 oscillations/min.

### Enzyme-Linked Immunosorbent Assay (ELISA)

To perform the assay, a fixative solution containing 1 µg/mL of SARS-CoV-2 RBD protein (GenScript) was prepared in carbonate-bicarbonate buffer (pH 9.6) and then a Nunc MaxiSorp flat bottom plate (Sigma) was coated with 100 µL of the fixative solution and incubated at 4°C overnight. The next day, the plate was washed five times with DPBS 0.05% (v/v) Tween-20 buffer (0.05% DPBS-T) and blocked with 3% (w/v) of skim milk (BD Biosciences) in 0.05% DPBS-T for 2 hours at room temperature, the plate was then washed five times with 0.05% DPBS-T. 100 µL of sera diluted 1/2000 (week 1 post-vaccination to week 7 post-vaccination) and 100 µL of purified total IgY antibodies (0.3 mg/mL) diluted 1/800 (week 1 post-vaccination to week 10 post-vaccination) both with 1% (w/v) of skim milk were added to the plate and incubated for 1 hour at 37°C. Later, wells were washed five times with 0.05% DPBS-T and immediately incubated with 100 µL of Goat anti-Chicken IgY secondary antibody conjugated with HRP (Genscript) diluted 1/2000 in 1% non-fat milk in 0.05% DPBS-T for 1 hour at 37°C. The plate was then washed five times with 0.05% DPBS-T and incubated with 100 µL of 3,3’,5,5’-tetramethylbenzidine (TMB) for 15 min at room temperature. The reaction was stopped by the addition of 50 µL of 2N H_2_SO_4_ per well, and the plate was read at 450 nm using an Epoch 2 microplate reader (Bioteck).

### Block of the RBD Binding to Vero E6 Cells Assay

After the highest immunoreactivity was found by ELISA, the corresponding IgY-R were subjected to a blocking assay to assess binding interactions of RBD to ACE2 protein on the Vero E6 cells surface. For this, Vero E6 cells were harvested and washed with FACS buffer. 1x10^6^ cells were blocked with FACS buffer with 5% of normal mouse serum for 30 min at 37°C. Then, the cells were incubated with a mix containing 8 mg/mL of purified IgY-R and 8 µg/mL of RBD (Sino Biological), for 2 hours at 37°C. To remove the IgY and RBD residual not attached to Vero E6, the cells were washed with FACS buffer twice. After, the mix was marked with rabbit monoclonal antibody anti-SARS-CoV-2 S1 (1:200) (Sino Biological) as the primary antibody for 1 hour at 37°C, followed by goat anti-rabbit IgG Alexa Fluor 488 (1:200) as the secondary (Abcam). Cells were acquired by the flow cytometer FACS Canto II (BD Biosciences), and data analyzed using the software FlowJo v.10.6 (BD Biosciences). Graphics were constructed using the software GraphPad Prism 8.0.1.

### Western Blot

To demonstrate the recognition activity of the viral RBD protein by the IgY-R pool, a Western Blot assay was carried out starting with an SDS-PAGE run of SARS-CoV-2 RBD protein (GenScript) at a rate of 0.3 µg per well, and the WB-MASTER Protein Standard (Genscript) as ladder, following the same procedure as described above. The content of the resulting gel was transferred to a nitrocellulose membrane using the eBlot L1 Protein Transfer System, according to the manufacturer’s recommendations. Then, the membrane was subjected to a 10-min wash with TBST wash buffer (tris-buffered saline and 0.1% Tween 20), and blocking was performed with PBS buffer supplemented with 0.1% Tween 20 and 3% milk, for 1 h. Subsequently, a wash step was performed, and the membrane was incubated with a dilution of IgY-R antibodies (1mg/mL) at a ratio of 2:5000 in the Azure Protein Free Blocking Buffer for 2 h. Then, another wash step was applied, and Goat anti-Chicken IgY secondary antibody conjugated with HRP (Genscript) at a ratio of 2:5000 in the Azure Protein Free Blocking Buffer was added, following incubation for 2 h, and a wash step prior to incubation with luminol (Azure Biosystems) for 2 min. Afterward, the membranes were revealed and photographed in a CCD camera (Azure Biosystems).

### SARS-CoV-2 Surrogate Virus Neutralization Test (sVNT)

IgY-R pooled samples were evaluated under the SARS-CoV-2 Surrogate Virus Neutralization Test (sVNT) (GenScript) to confirm the ability to block the binding of RBD to ACE2 receptor, following the manufacturer’s instructions. Briefly, 60 µL of 0.3 mg/mL IgY-R sample were mixed 1:1 with properly diluted HRP conjugate RBD and incubated for 30 min at 37°C, then 100 µL of the mix was added to the 96-well flat-bottom plate containing the ACE2 protein and was incubated for 15 min at 37°C. Later, three washing steps were performed, and 100 µL of TMB solution was added to each well and the plate was incubated for 15 min. The reaction was stopped with 50 µL of stop solution and the plate was read on the EPOCH-2 spectrophotometer (Biotek) at a wavelength of 450 nm. Positive and negative controls were diluted 1:10 with sample dilution buffer.

### IgY-R Safety Studies in Mice

For the *in vivo* safety evaluation of IgY-R, 3 experimental groups were considered (n=5 per group), two of them receiving either 30 µg of IgY-R per gram of body weight (3X dose) or PBS (mock) intranasally, and an additional untreated group (blank). Mice were monitored daily for clinical signs and mortality for 3 weeks, prior to being humanely sacrificed, using an overdose of a mixture containing Ketamine (100 mg), Xylazine (20 mg), and Atropine sulfate (1 mg) (Agrovet), intraperitoneally. Right lung, trachea, liver, gut, and kidney samples were collected and fixed in 10% buffered formalin for 48 h prior to being processed for paraffin embedding. The tissue was cut to a thickness of 5 µm and stained with hematoxylin and eosin (H&E) for histopathological assessments, using an AxioCam MRc5 camera and an AxioScope.A1 microscope (Carl Zeiss) at 20x and 40x magnification, performed by a certified veterinary pathologist in a blinded manner.

### Kinetics of Intranasally Delivered IgY-R

The persistence kinetics of IgY-R when intranasally delivered were evaluated at the local level in the oropharyngeal region and at the systemic level in serum. For this, six hamsters were inoculated with a dose of 10 µg of IgY-R per gram of body weight (1X), intranasally, and four hamsters with PBS for the control group. Subsequently, oropharyngeal swab and blood samples from gingival vein were taken at 24 and 48 h after the administration of IgY-R and processed by soaking swabs into 100 µL of PBS, allowing blood to coagulate at room temperature for 1 h, then centrifuging at 5000 g for 5 min to obtain serum. The solution obtained from swabs and serum were diluted 1:2 and 1:50 with 1% (w/v) of skim milk, respectively, and subjected to ELISA assay similar to that described above, incubating samples at 37°C in a plate previously fixed with RBD protein and blocked with skim milk, prior to incubation with Goat anti-Chicken IgY secondary antibody conjugated with HRP (Genscript) diluted 1:20000, at 37°C for 1 h for the subsequent TMB addition and reading at 405 nm.

### SARS-CoV-2 Challenge in Hamster

To test the efficacy of IgY-R, acclimatized hamsters were distributed in four groups of four animals each (two males and two females per group), corresponding to the prophylactic antiviral treatment (PAT), post-infection treatment (PIT), and infection without treatment (Control). Animals within the PIT, PAT, and control group were transferred to the BSL3 and challenged intranasally with 10^5^ PFU of SARS-CoV-2, in a volume of 40 µL. The administration of IgY-R was carried out in doses of 40 µL, at a concentration of 1X, also intranasally. For the PAT group, a single dose was administered 2 h prior to infection with the SARS-CoV-2 virus, while for the PIT group, a total of three doses were administered, corresponding to 2-, 24-, and 48-hours post-infection.

### Body Weight Variation Assessment

The analysis of the percentage variation of body weight was based on the collection of weight data of hamsters within the challenge study at 0-, 24-, 48-, and 72-h post-infection with SARS-CoV-2. The percentages of change with respect to the initial weight of the individuals were calculated and plotted with the GraphPad Prism 8.0.1 software.

### Animal Mobility Evaluation

The average speed, average acceleration, and average displacement were calculated, based on videos recorded with a camera positioned on top of the cages up to 3 days post-infection (DPI) of hamsters with SARS-CoV-2. The conditions of video filming (distance, focus) were always the same, so the pixels always reflect the same spatial separation. Hamsters were tracked in the time intervals in which they were moving away from the edge of the box, using the Kernelized Correlation Filter (KCF) algorithm through the OpenCV library and the Python language. Graphs were generated using matplotlib.

### Tissue Protection

To test tissue protection from SARS-CoV-2, a procedure similar to that used in safety studies was applied, in which hamsters from all groups were humanely sacrificed at 3 DPI prior to collecting and fixing right lung and trachea samples for subsequent staining with H&E.

### Statistical Analysis

Results are expressed as mean ± SD. Cutoff values were established following the t-student distribution approach (31) for ELISA assays, with a CI of 95%. Weight changes were analyzed under the Tukey test, with a CI of 95%. For mobility assessments, Mann-Whitney and Kruskal-Wallis non-parametric tests were performed comparing PIT and PAT groups with the control group. A P value of < 0.05 was considered statistically significant. Asterisks (*), (**),and (***) stands for P < 0.05, 0.005, and 0.0005, respectively. Error bars represent SD.

## Results

### IgY-R Isolation and Purity

**Figure 1:**
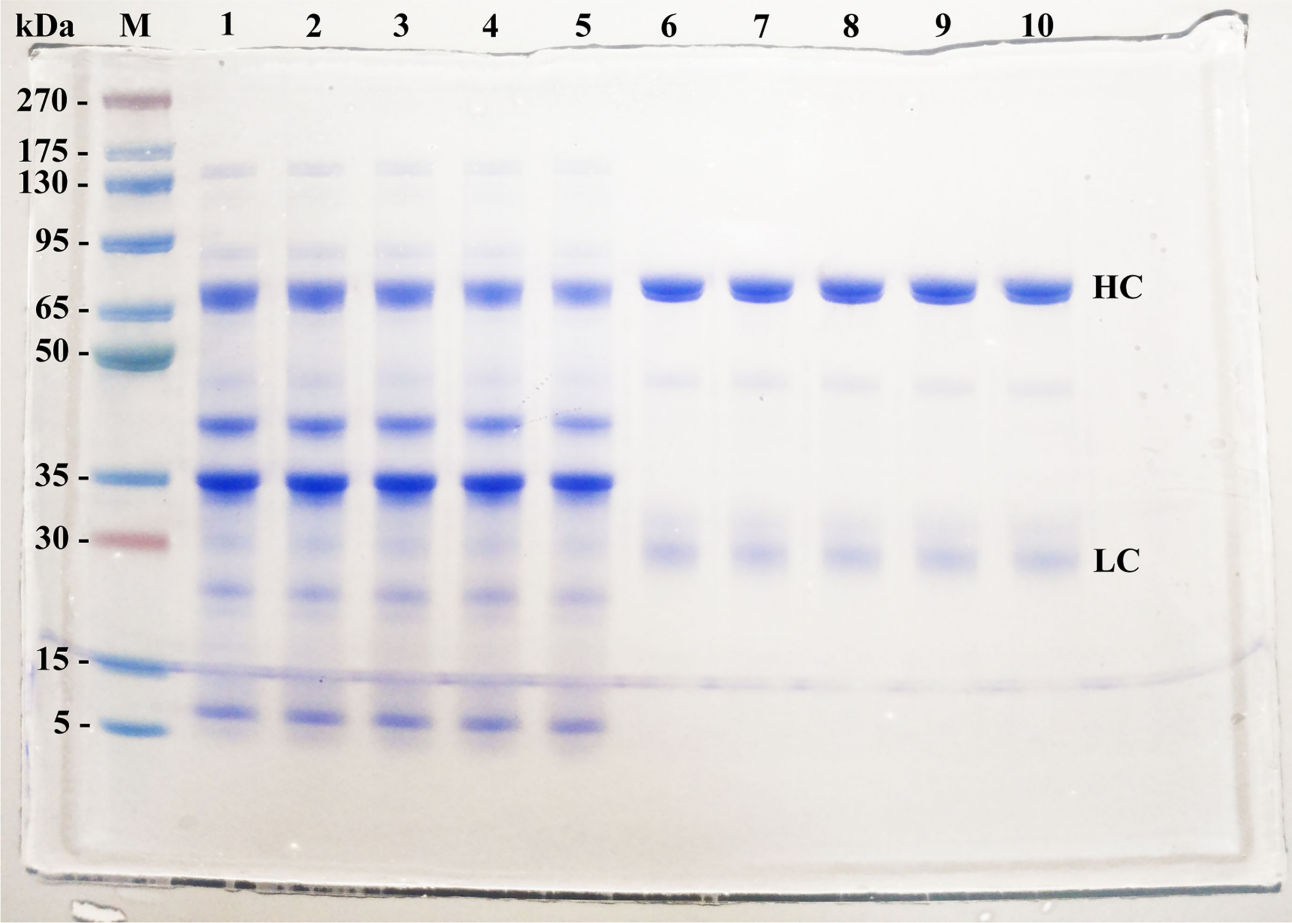
SDS-PAGE patterns of IgY-R at the 2 stages of purification (4 µg per line). M stands for molecular weight marker, with size indicated on the left; Lanes 1-4: WSF of yolks belonging to hens immunized with 5, 12.5, 25, and 50 µg of rRBD; lane 5: WSF of control yolks; lanes 6-9: purified IgY of yolks belonging to hens immunized with 5, 12.5, 25, and 50 µg of rRBD; lane 10: purified control IgY. HC, Heavy chain; LC, Light chain.

The mean yield obtained was 4.9 mg of IgY per mL of egg yolk. IgY purity reached 96%, as indicated by SDS-PAGE. Protein bands were shown with a pattern in accordance with previous data reported, consisting of two major protein bands of ≈68 kDa and ≈27 kDa corresponding to heavy and light chains of IgY-R, respectively (**Figure 1**).

### IgY-R *In Vitro* Immunoreactivity

**Figure 2:**
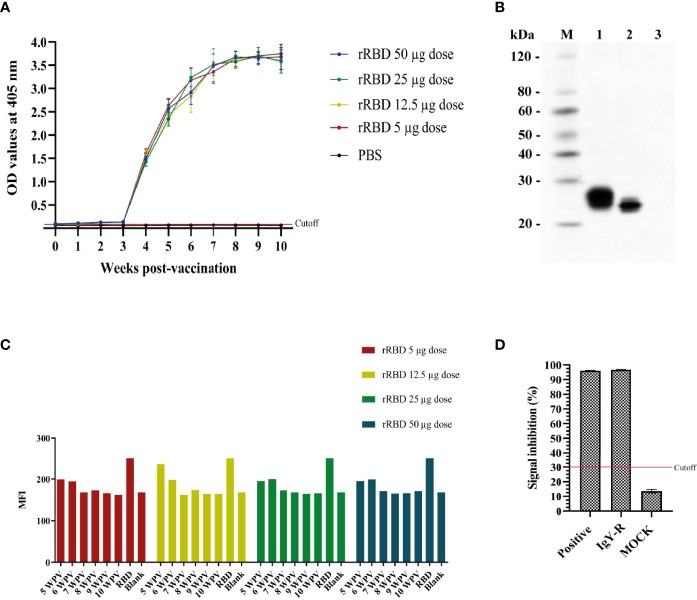
*In vitro* immunoreactivity of IgY-R. **(A)** IgY-R raising pattern according to ELISA against the SARS-CoV-2 rRBD antigen both in serum and egg yolks of immunized hens. **(B)** Western Blot using IgY-R as primary antibody. Lane M: Ladder; lane 1: reduced RBD; lane 2: non reduced RBD; lane 3: PBS as control. **(C)** IgY-R ability to block RBD binding to Vero E6 cells. Results are presented as the mean fluorescence intensity (MFI) given by the flow cytometer **(D)** Neutralization percent of IgY-R using the cPass SARS-CoV-2 Neutralization Antibody Detection Kit. Cutoff value is settled as 30% following manufacturer guidelines. Mean ± SD are presented.

It was shown through ELISA that immunoreactivity against RBD protein of IgY-R started to raise after 1 WPV and 3 WPV for serum and egg yolks samples, respectively, and sustained a similar pattern throughout the 10 weeks evaluation (**Figure 2A**). Regarding the Western Blot assay, RBD protein was recognized under reducing and non-reducing conditions by IgY-R, while no visible band was shown in the control line (**Figure 2B**). For the binding assay, cells incubated with IgY isolated from hens immunized either with 5, 12.5, 25, or 50 µg of rRBD presented less MFI compared with cells incubated only with rRBD, which indicates that the percentage of RBD bound to Vero E6 cells is lower (**Figure 2C**). Additionally, IgY-R was able to recognize RBD protein under reducing and non-reducing conditions, with a negative result using BSA as control, also showing a neutralization percent nearly to 100%, similarly to the positive control, under the Neutralization Antibody Detection Kit (**Figure 2D**).

### Safety

**Figure 3:**
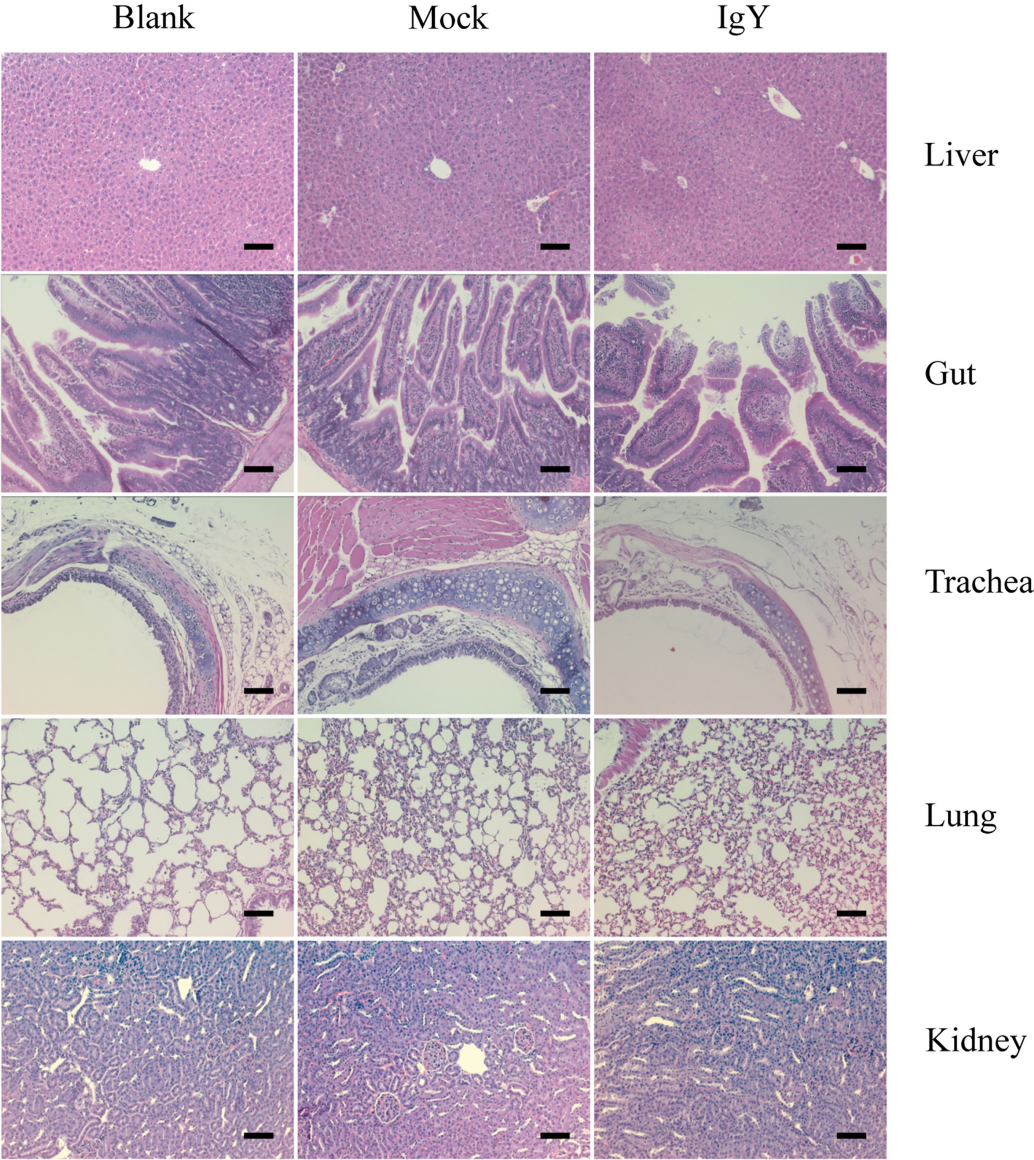
Safety of IgY-R through histopathological assessment of liver, gut, trachea, lung, and kidney of mice. No visible lesions were found on any of the slides analyzed (six slides per tissue sample). Representative images are shown. Image amplitude: 20x Scalebars: 200 µm.

The histopathological evaluation showed no visible pathological signs in any of the groups considered, as well as no signs of morbidity and no mortality, demonstrating the safety of intranasal administration of IgY-R under the proposed scheme of three doses at 30 μg/g (**Figure 3**).

### Kinetics of IgY-R

**Figure 4:**
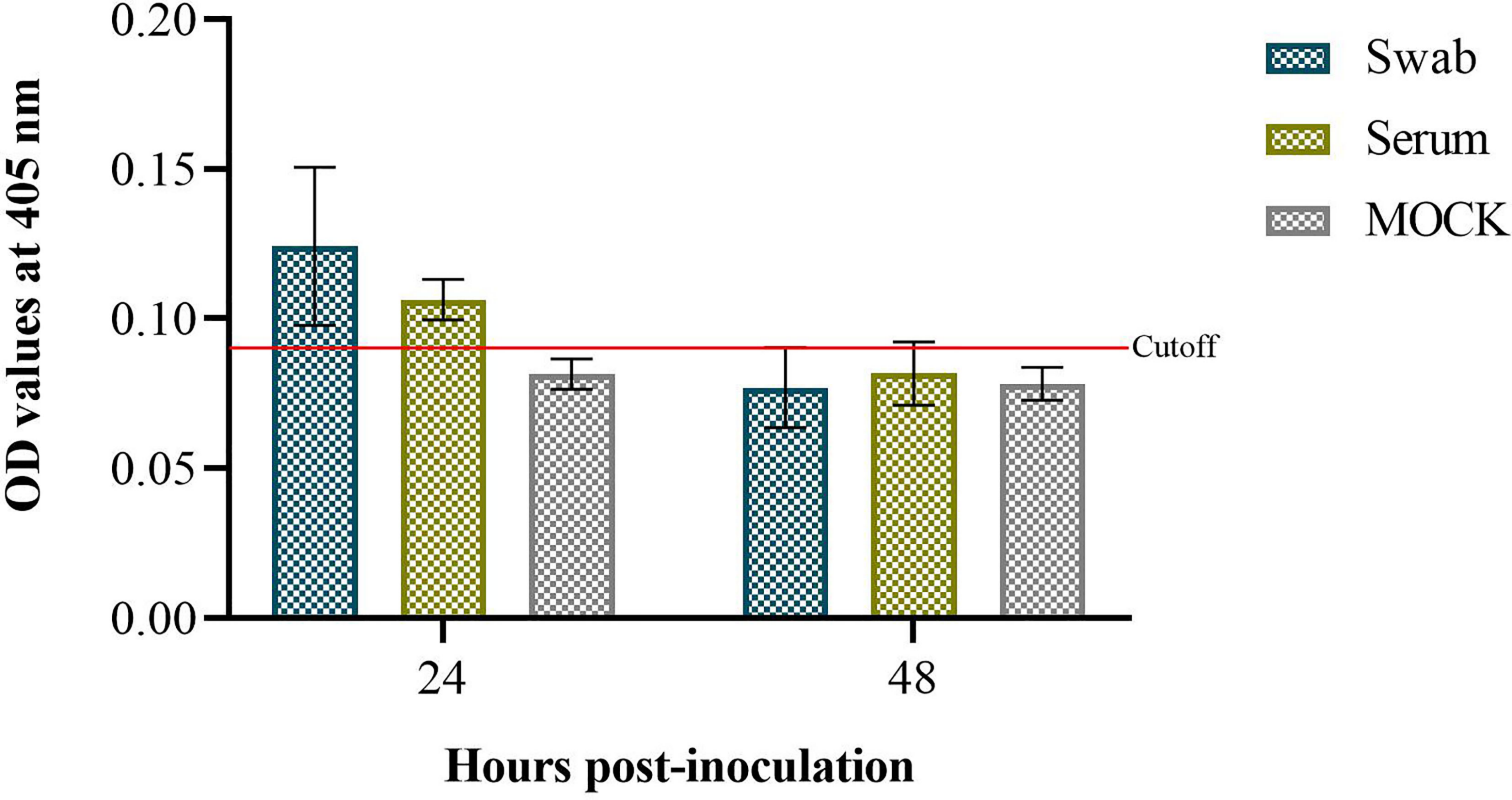
Assessment of intranasally delivered IgY-R in hamster recovered from serum and nasal swabs. Cutoff value was set to 0.10 (CI = 95%). Mean ± SD (error bars) is presented (n = 4 per group).

The results of the persistence of IgY-R after intranasal administration were positive 24 h after administration, being higher locally than systemically. Analysis 48 h after administration was negative locally and systemically (**Figure 4**).

### Efficacy

**Figure 5:**
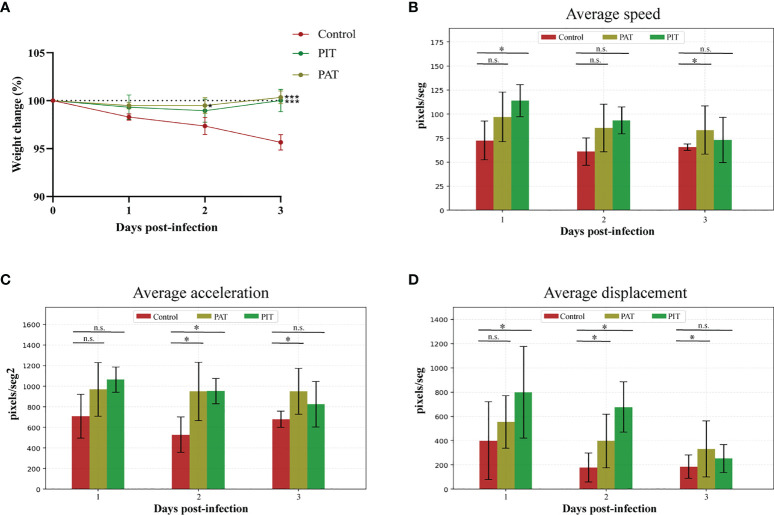
Physical outcomes evaluation of hamsters infected with SARS-Cov-2 and intranasally administered with IgY-R. **(A)** Percentage variation of the weight of individuals over the experimental time-course. **(B)** Average speed variation **(C)** Average acceleration variation **(D)** Average displacement variation. DPI = Days Post Infection. Mean ± SD (error bars) is presented (n = 4 per group). The Mann-Whitney and Kruskal Wallis tests were performed to determine whether differences between Control and IgY-administered groups were significant (*) or non-significant (n.s.).

**Figure 6:**
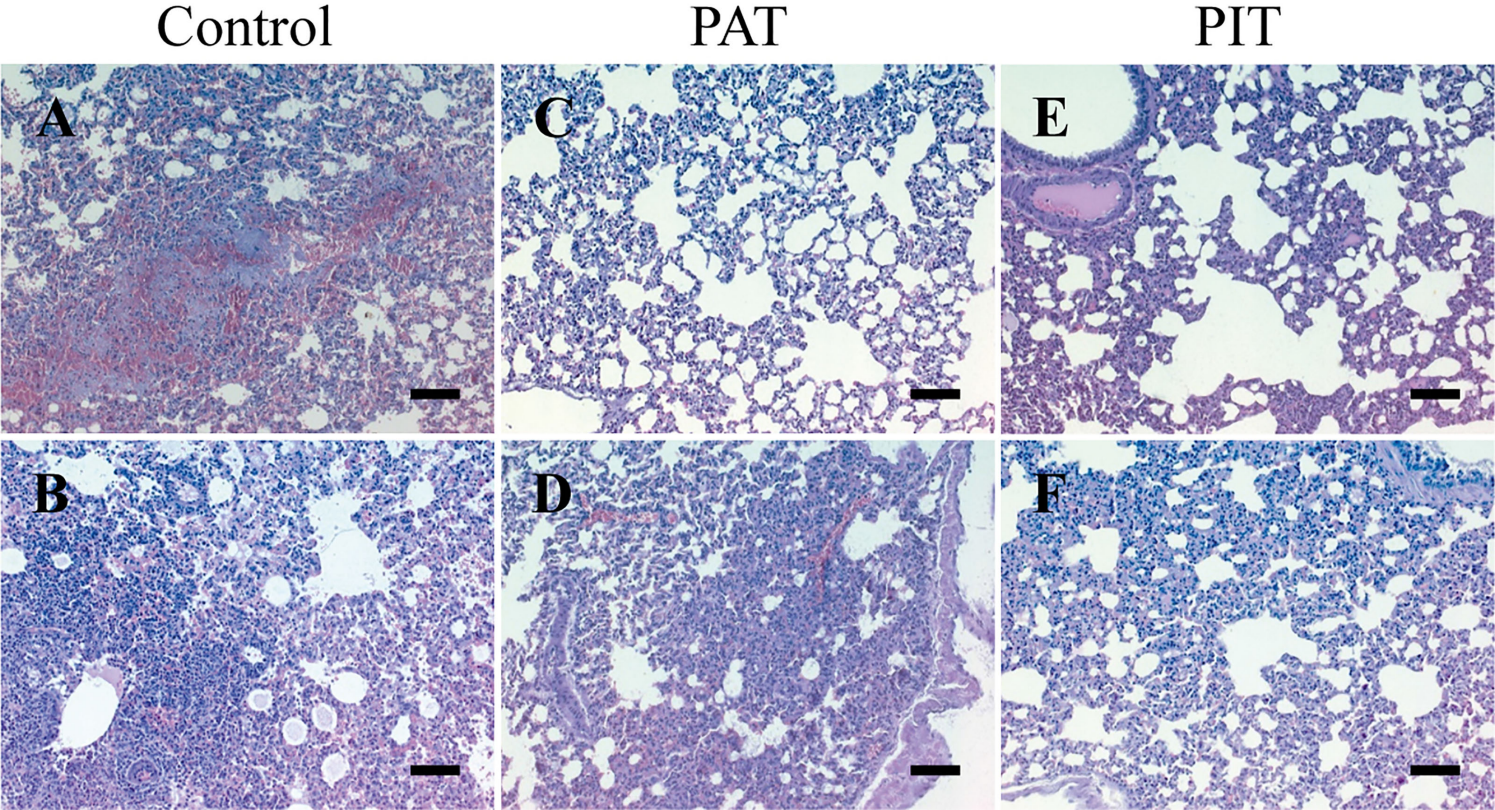
Histopathological findings in the lungs of hamsters challenged with SARS-CoV-2. **(A)** Areas of necrosis with hemorrhage, thickened alveolar wall, and infiltration mononucleated inflammatory cells **(B)** Invasion of intraalveolar mononuclear inflammatory cells **(C)** No visible lesions **(D)** The pulmonary parenchyma appears thickened in some areas with atelectasis, the alveoli are clean, there is no infiltration of inflammatory cells **(E)** Slightly thickened pulmonary parenchyma, the alveoli slightly dilated, but without infiltration of inflammatory cells **(F)** Thickening of the alveolar wall, the alveolar lumen does not present cellular infiltration. Image amplitude: 20x. Scalebars: 200 µm.

Initial weight was not significantly different in any of the experimental groups before challenge, and there was no significant change in weight percentage between treatments until 3 DPI, in which PIT and PAT groups differed significantly from control (**Figure 5A**). Regarding kinematics, all parameters measured were significantly different comparing PIT and control groups by 3 DPI (**Figures 5B, C, D**). H&E staining of the lungs at 3 DPI detected bronchopulmonary hemorrhage and pneumonia for the control group, in contrast to the observations of the PIT and PAT groups, which showed no apparent visible lesions, except for an observation of atelectasis. The rest of the sections of the latter only present a thickening of the parenchymal alveolar wall (**Figure 6**).

## Discussion

Due to the high rate of transmission of SARS-CoV-2 virus, the COVID-19 pandemic represents a significant challenge for health systems worldwide (32). Furthermore, the possibility that new variants of the virus will appear that escape the antibodies generated by the vaccines puts the efficacy of this measure at an uncertain level (33, 34). In this sense, it is necessary to develop a countermeasure of rapid production, high yield, and low cost so it can be quickly updated against multiple variants of the virus if necessary and be applied in those countries with a higher incidence of cases, which coincide with impoverished human populations (35).

In the present study, IgY antibodies directed to the RBD domain of SARS-CoV-2 were generated, and its ability to inhibit the interaction with the ACE2 receptor was demonstrated. Furthermore, these antibodies were effective in reducing the physiological stress and the histopathological damage in the lungs of challenged hamsters.

Previous studies have proposed chicken-IgY antibodies as a possible therapeutic agent against COVID-19 disease (26, 36–38), wherein *in vitro* and *in vivo* studies producing IgY against inactivated virus (39), Spike protein (40–43), or subunit S1 (44) have demonstrated promising results, using 50 µg of antigen in a three-dose immunization scheme and isolating IgY following the PEG method (45).

In comparison, our results showed that using a vaccination strategy of three doses of a recombinant SARS-CoV-2 antigen in combination with a poultry adjuvant every 2 weeks it is possible to obtain IgY antibodies with high recognition and neutralization activity. This is likely to be because of the antigenic anatomy of SARS-CoV-2 RBD, carrying the most immunogenic epitopes against which most neutralizing antibodies are generated (46). Besides, the use of a biocompatible method that does not require potentially harmful reagents or expensive materials also provides antibodies with a high purity/yield (**Figure 1**) of which 2 to 10% are known to be specific antibodies (47).

In terms of the production of IgY, the use of hens for the generation of antibodies has several economic advantages over the use of other animals. For example, hen keeping costs are lower than those of mice and rabbits; the amount of antibodies produced by chickens also corresponds to that of larger animals, such as goats and sheep, reducing the number of animals needed for antibody production (48). Moreover, a hen lays about 300 eggs and produces an average of 18.25 g of IgY in a year (45).

Among hens, SPF hens can generate antibodies with a high concentration, high yield, and absence of specific pathogens (49). However, it is more common to obtain IgY by inoculating laying hens on sheds (23, 39–41, 50–54) Therefore, future improvements may include the use of laying hens as this can reduce even more the costs of producing IgY.

Another relevant finding concerning costs is that antibody levels were mostly the same regardless of the dose of antigen used when testing from 5 to 50 ug of rRBD (**Figure 2A**). According to previous reports, at doses between 10-100 ug of antigen per individual, adequate levels of specific IgY are obtained, under ELISA assay (25, 55, 56). Moreover, IgY antibody response to doses in the range of 1 to 100 ug of bovine serum albumin was proven to be similar from the sixth week after prime dose (57). Likewise, human IgG antigen doses of 2 ug, 20 ug, and 200 ug increased antibody levels in a similar way after the third immunization, supporting the importance of testing different antigen amounts to improve the cost-effectiveness of the method (58).

Pharmacokinetic studies demonstrated the presence of IgY during the first 24 h (39), proving that IgY can remain in the upper airways for a few hours, and serve as a prophylactic, which is consistent with our results (**Figure 4**). On the other hand, there is a minimal diffusion of IgY through the nasal mucosa as occurs with low molecular weight drugs, due to low mucous membrane permeability and the presence of proteolytic enzymatic activity in the nasal mucosa, leading to a low bioavailability for hydrophilic peptides and proteins (59), in contrast with immediate bioavailability when an intravenous administration is performed (**Supplementary Figure S1**), suggesting the need to administer multiple intranasal doses at high concentration to obtain a beneficial effect.

In order to improve IgY absorption and its use as a post-infection treatment, there are multiple approaches, including enhancers (60) such as bile salts and surfactants facilitating diffusion through the nasal mucosa or the use of a carrier system like liposomes, nanoparticles, and microparticles (61–63).

Otherwise, passive immunization using IgY antibodies has proven to be a safe alternative as was revealed by histopathological assessments (**Figure 3**). Along with this, it does not bind to human Fc receptors or react with the complement system, so the risk of inflammation and dangerous immune responses is minimal (64) and it has a higher specificity and target binding strength than IgG immunoglobulin. Besides, IgY extracts are considered well tolerated because chicken eggs are part of the human diet and they can be used considering that purified IgY is devoid of albumin, a common trigger of allergic reactions present in egg white (65).

Nonetheless, serum sickness is still a theoretical possibility if IgY is administered in large amounts, given that antigenicity has been previously verified in pigs and mice (54, 66), thus, multiple administrations are preferred instead of a single injection when a high dose is required (67). Furthermore, IgY extracts may carry chicken allergens present in the yolk as contaminants, such as egg yolk alpha-livetin, also known as Gal d5 allergen, a thermolabile protein that could lead to the bird-egg syndrome, which is characterized by respiratory and gastrointestinal symptoms including asthma and rhinoconjunctivitis (68).

Within *in vivo* experiments, hamsters treated before and after the infection with SARS-CoV-2 showed a visible reduction in lung pathology compared to untreated animals (**Figure 6**). These findings indicate that IgY-R has a protective efficacy both as a prophylactic or a post-infection treatment when intranasally delivered, similar to that reported in previous studies using prophylactic IgY against influenza viruses (23, 69, 70) or as a post-infection treatment against psoriasis, gluten-related allergies (62), Helicobacter pylori (71,) or Ebolavirus (67), in which the route of administration was oral or intraperitoneal.

Regarding the suitability of the intranasal route, this relies on the nasal mucosa structure, which is a rather porous and thin endothelial basement membrane when compared to other biological membranes. It also has rapid blood flow, with a highly vascularized epithelial layer and a large absorption area. These characteristics give many advantages such as a very fast absorption and rapid action of drug (72, 73). Interestingly, the most common route of entry of SARS-CoV-2 is the respiratory tract, which makes appropriate the use of intranasal drugs as protection at a local level.

Although this study has certain limitations, such as the number of individuals immunized to obtain IgY and hamsters within the challenge assay, in addition to the fact that further studies are required to determine the most suitable approach to overcome certain allergenicity issues, our results were homogeneous and consistent with previous data reported, showing great potential to be used as a base for future studies aiming the conversion of IgY products into clinical practice against SARS-CoV-2.

## Covid 19 Working Group In PerÚ Members

Andres Agurto-Arteaga, Ricardo Antiparra, Manuel Ardiles-Reyes, Katherine Calderón, Yudith Cauna-Orocollo, Maria de Grecia Cauti-Mendoza, Naer Chipana-Flores, Ricardo Choque-Guevara, Xiomara Chunga-Girón, Manuel Criollo-Orozco, Lewis De La Cruz, Nicolás E. Delgado-Pease, Elmer Delgado-Ccancce, Christian Elugo-Guevara, Manolo Fernández-Díaz, Manolo Fernández-Sánchez, Luis Guevara-Sarmiento, Kristel Gutiérrez, Oscar Heredia-Almeyda, Edison Huaccachi-Gonzalez, Pedro Huerta-Roque, Eliana Icochea, Gisela Isasi-Rivas, Gabriel Jiménez-Avalos, Romina A. Juscamaita-Bartra, Abraham Licla-Inca, Angela Montalván, Ricardo Montesinos-Millán, Dennis Núñez-Fernández, Adiana Ochoa-Ortiz, Gustavo E. Olivos-Ramirez, Erika Páucar-Montoro, Kathy Pauyac, Jose L. Perez-Martinez, Norma Pérez-M, Astrid Poma-Acevedo, Stefany Quiñones-Garcia, Ingrid Ramirez-Ortiz, Daniel Ramos-Sono, Angela A. Rios-Angulo, Dora Rios-Matos, Aldo Rojas-Neyra, Yomara K. Romero, Mario I. Salguedo-Bohorquez, Yacory Sernaque-Aguilar, Patricia Sheen-Cortavarría, Luis F. Soto, Luis Tataje-Lavanda, Julio Ticona, Katherine Vallejos-Sánchez, A. Paula Vargas-Ruiz, Doris Villanueva-Pérez, Freddy Ygnacio-Aguirre & Mirko Zimic-Peralta.

## Data Availability Statement

The raw data supporting the conclusions of this article will be made available by the authors, without undue reservation.

## Ethics Statement

The animal study was reviewed and approved by Bioethics Committee of the Universidad Nacional Hermilio Valdizán. Written informed consent was obtained from the owners for the participation of their animals in this study.

## Author Contributions

MFD, MZ, LG, AA-A, and AP-A conceived the study. AA-A, AP-A, YC-O, and SQ-G designed experiments under the supervision of MFD, MZ, and LG. AA-A, AP-A, DR-M, AM, GI-R, MC-M, NP-M, and IR-O performed experiments. RC-G, RM-M, IR-O, and KG-M provided the recombinant RBD protein for the study. DN-F and MS-B performed kinematics assessments. AA-A, AP-A, and DR-M analyzed and interpreted the data. AA-A and MZ received data and organized the structure of the paper. The paper was written by AA-A, AP-A, DR-M, RC-G, and RM-M. The COVID-19 Working Group in Perú provided bibliographic assistance and logistic support. All authors read and approved the final manuscript.

## Funding

This research was partially supported by the “Fondo Nacional de Desarrollo Científico, Tecnológico y de Innovación Tecnológica” (FONDECYT) under the agreement 080-2020-FONDECYT.

## Conflict of Interest

The authors AA-A, AP-A, DR-M, RC-G, RM-M, AM, GI-R, MFD and LGS are employed by FARVET SAC. Author MZ is a consultant for company FARVET SAC. The authors MC-M, NP-M, KG-M & IR-O were employed by FARVET SAC at the time the study was conducted.

The remaining authors declare that the research was conducted in the absence of any commercial or financial relationships that could be construed as a potential conflict of interest.

## Publisher’s Note

All claims expressed in this article are solely those of the authors and do not necessarily represent those of their affiliated organizations, or those of the publisher, the editors and the reviewers. Any product that may be evaluated in this article, or claim that may be made by its manufacturer, is not guaranteed or endorsed by the publisher.
