# Supplementary material for: Preclinical Assessment of IgY Antibodies Against Recombinant SARS-CoV-2 RBD Protein for Prophylaxis and Post-Infection Treatment of COVID-19
Source: Front Immunol. 2022 May 10;13:881604. doi: 10.3389/fimmu.2022.881604 (PMC9157249; doi:10.3389/fimmu.2022.881604)
Supplement: Supplementary file 1 [file DataSheet_1.docx]

Supplementary Material


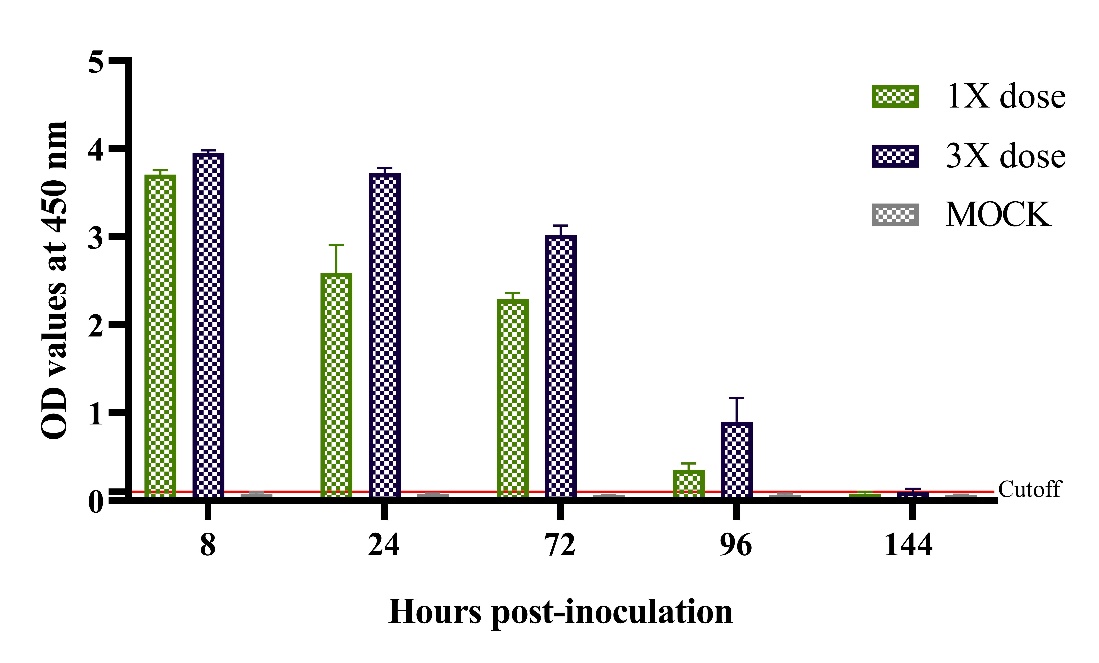
For the evaluation of immediate bioavailability at the systemic level of IgY-R, an intravenous administration of IgY-R was assayed. Mice were distributed in three groups of 6 individuals each, including a group receiving a 1X dose of IgY-R, another receiving a 3X dose of IgY-R, and a group that receives PBS. Blood samples were taken by puncture of the submandibular vein and processed, allowing blood to coagulate at room temperature for 1 h, then centrifugating at 5000 g for 5 min to obtain serum. The solution was diluted 1:50 with 1% (w/v) of Skim Milk and subjected to ELISA assay incubating samples at 37° C in a plate previously fixed with RBD protein and blocked with Skim Milk, prior to an incubation with Goat anti-Chicken IgY secondary antibody conjugated with HRP (Genscript) diluted 1:20000, at 37°C for 1 hour for the subsequent TMB addition and reading at 405 nm. Active IgY-R were found in mice sera from 8 to 96 hours after intravenous treatment was applied, with a sustained significant difference between 1X and 3X doses within these analysis points. At 144 hours post-inoculation, the mean of both treatments was below the cutoff value.

**Supplementary Figure 1. Bioavailability of IgY-R administered intravenously in mice**. Cutoff value was set to 0.10 (CI = 95%). Mean ± SD are presented (n = 4 per group).
